# Simultaneous Treatment of Osteochondral Lesion Does Not Affect the Mid- to Long-Term Outcomes of Ligament Repair for Acute Ankle Sprain: A Retrospective Comparative Study with a 3–11-Year Follow-up

**DOI:** 10.3389/fsurg.2022.816669

**Published:** 2022-05-09

**Authors:** Ming-Ze Du, Tong Su, Yan-Fang Jiang, Chen Jiao, Qin-Wei Guo, Yue-Lin Hu, Dong Jiang

**Affiliations:** ^1^ Department of Sports Medicine, Peking University Third Hospital, Beijing, China; ^2^ Institute of Sports Medicine of Peking University, Beijing Key Laboratory of Sports Injuries, Beijing, China

**Keywords:** acute ankle sprain, osteochondral lesion, arthroscopy, return to play, lateral ankle ligament

## Abstract

**Purpose:**

This study aims to evaluate the mid- to long-term outcome of concurrent arthroscopic treatment of osteochondral lesion (OCL) and open anatomical repair of lateral ankle ligaments for severe acute ankle sprain patients and compare them to the outcome of those without OCL.

**Methods:**

A total of 166 patients with grade III acute lateral ankle ligament injuries underwent concurrent ankle arthroscopy and open anatomic ligament repair. Forty-three patients (group A) with OCL underwent arthroscopic treatment followed by open ligament repair. A total of 105 patients (group B) without OCL were followed up as the control. The evaluation parameters included sports recovery, postoperative visual analog scale (VAS) pain score, American Orthopaedic Foot and Ankle Society (AOFAS) score, Tegner score, sprain recurrence, satisfaction, and range of motion. Patients in group A were then subgroup-analyzed according to age, sex, body mass index, injury side, OCL location, and stage (Ferkel and Cheng’s staging system).

**Results:**

The postoperative exercise level of the two groups recovered to more than 90% of the normal level (91.2% ± 11.2% in group A and 90.9% ± 13.3% in group B, n.s.). The average time of group A and group B to return to preinjury sports activity was respectively 4.4 ± 1.0 months and 4.4 ± 1.2 months with no significant difference (*p* = 0.716). No significant differences were found in the preoperation VAS pain score, AOFAS score, and Tegner score between the two groups. The postoperative VAS pain score in group A was significantly higher than that in group B (0.8 ± 1.7 vs. 0.3 ± 0.8, *p* = 0.027), but the difference was not clinically important. The postoperative VAS pain score of patients with stage D–F lesions was significantly higher than that of patients with stage B–C lesions (1.3 ± 2.1 vs. 0.3 ± 0.9, *p *= 0.038).

**Conclusions:**

For the severe acute ankle sprain combined with OCL, the simultaneous arthroscopic treatment and open lateral ankle ligament repair achieved good mid- to long-term outcomes. Except that the pain was more pronounced than in the control group, there were no differences in other outcomes. Postoperative pain was positively correlated with the grade of OCL.

## Introduction

Ankle sprains were reported to account for more than 10% of all sports injuries, among which lateral ligament sprain was the most common (1, 2). Rehabilitation with optimal loading in a brace was advocated for most ankle sprains (3). Ankle ligament sprains are usually graded on the basis of the severity of ligament rupture. For patients with grade III ankle ligament (4) and high demand for sports, conservative treatments showed a lower satisfaction rate and surgery is more recommended (5, 6).

The previously reported incidence of osteochondral lesion (OCL) combined with acute ankle sprain was up to 95% (7–10). If not properly treated, the OCL will induce a cascade of events, with the potential of leading to end-stage osteoarthritis, either localized or of the whole joint (11). The surgical treatment for the ankle OCL included abrasion, debridement, microfracture, drilling, and autograft or allograft transplantation, in which bone marrow stimulation (BMS) was the most commonly used. These surgical treatment procedures aim to regenerate tissue similar to natural cartilage, provide symptomatic relief, and return the patient to sports (12, 13). However, excessive intra-articular trauma caused by BMS will cause relatively more bleeding and inflammation, which may lead to complications such as limited joint movement, which has been reported to negatively affect the effect of ligament repair surgery for chronic ankle instability (CAI) (14–16).

Compared to CAI, the OCL with acute ankle sprain contained more osteochondritis dissecans, tangential fractures, and bone marrow edema but fewer subchondral bone cysts and combined joint degeneration (17, 18). These different characteristics of OCL may have different effects on clinical outcomes. So far, there has been no study on the impact of the simultaneous treatment of OCL on the long-term outcome of ligament repair for severe acute ankle sprains.

In the present study, all the acute ankle sprain patients with simultaneous grade III ligament rupture and OCL undergoing concurrent arthroscopy and open ligament repair between 2007 and 2017 in our institute were followed up. Patients with isolated arthroscopic exploration and acute ligament repair during the same period served as controls. The purpose of this study was to evaluate the mid- to long-term outcome of concurrent arthroscopic OCL treatment and open anatomical repair of lateral ankle ligaments of acute ankle sprain and compare them to the outcome of those without OCL. It was hypothesized that simultaneous treatment of OCL does not affect the mid- to long-term outcomes of open ligament repair for acute ankle sprains.

## Materials and Methods

### Patient Selection

Most acute ankle sprain patients were recommended conservative treatments in our institute. For patients with grade III lateral ankle ligament rupture and high demand for sports, the option of surgical treatment was given. After being fully informed of the risk of surgery, patients giving consent were then admitted for surgery. All patients with acute lateral ankle ligament rupture (less than 2 weeks) who underwent arthroscopic exploration and open anatomical ligament repair from June 2007 to May 2017 were enrolled. The ethics license was obtained from the IRB Medical Committee (IRB00006761-2016011).

Patients were excluded if (1) the OCL area was greater than 15 mm^2^ or the depth of OCL was greater than 8 mm, requiring osteochondral transplantation rather than BMS and (2) they had index ankle surgery.

### Surgical Technique

Under general or spinal anesthesia, all patients underwent arthroscopic exploration and necessary treatment of intra-articular lesions before ligament repair. The location of talar cartilage injury is recorded according to the nine-zone method (19). OCL was measured using labeled probes and classified according to Ferkel and Cheng’s classification (20) in group A: stage A: smooth, intact but soft or ballotable; stage B: rough surface; stage C: fibrillation/fissuring; stage D: flap present or bone exposed; stage E: loose, undisplaced fragment; and stage F: displaced fragment. Rough or fibrotic cartilage debridement was performed in patients with stage B–C lesions using a mechanical razor system (Dyonics Power Shaver System; Smith & Nephew, Andover, MA, USA). For D–F stage lesions, in addition to debridement of pterygoid cartilage, BMS was performed, including abrasion, scraping, and microfracture. Other lesions (such as synovial hyperplasia, loose body, etc.) are also treated under arthroscopy. Similarly, for patients in group B, these intra-articular injuries were also explored under arthroscopy before open anatomical ligament repair.

After arthroscopic surgery, a slightly curved longitudinal incision was made 3–4 cm above the distal fibula to expose anterior talofibular ligament (ATFL) and calcaneofibular ligament (CFL). Care was taken to protect the dorsal cutaneous nerve and the sural nerve during the incision. The modified Broström–Gould technique (21) was used for anatomical repair of the lateral ankle ligament, and suture anchors were used for the insertion site rupture. One or two 5-mm-deep holes were drilled at the ruptured insertion position of ATFL or CFL, and 1.8-mm-diameter suture anchors (Mitek Mini GII; Johnson & Johnson, NJ, USA) were placed to fix the ligaments. Then, the extensor retinaculum was sutured to the fibular periosteum. The anterior drawer test and talus tilt were evaluated again to ensure sufficient stability of the ankle.

### Postoperative Rehabilitation

The splint was used for all patients 3 weeks after the operation without weight-bearing. Then, the splint was replaced with an ankle brace, and range of motion (ROM) training was performed until week 6. For group A patients, the splint or brace was removed daily from the second week to the sixth week for full-range continuous passive exercise (CPM) training. CPM exercise was performed in a painless range for 30 min a day. Then, reposition the splint to secure the ankle. Varus movement is allowed from week 5. Partial weight-bearing was allowed at 6–8 weeks, and complete weight-bearing was allowed at 8–12 weeks. For patients in group B, ROM training of flexion and extension was started from the third week. Complete weight-bearing was allowed at 4–6 weeks. All the patient resumes exercise according to the patients’ tolerance and recovery.

### Clinical Outcome Evaluation

The sports recovery and complications of all patients were recorded and evaluated. During the follow-up, if the sports returned to more than 85% of the preinjury level, it was recorded as returning to the preinjury sports. Assessments at the final follow-up included visual analog scale (VAS) pain score (22), American Orthopaedic Foot and Ankle Society (AOFAS) score (23), Tegner activity score (24), sprain recurrence, ROM, recovery of preinjury sports level, patient satisfaction, skin numbness area, and other complications. For scores that differ between the two groups, a subgroup analysis of group A was performed for the potentially related factors, including age, gender, body mass index (BMI), OCL side, injury zone, and OCL staging.

### Statistical Analysis

SPSS statistical software version 23.0 (IBM; Armonk, New York, USA) was used to analyze the data. The chi-square test or Fisher’s exact probability test was used for categorical results, while the paired sample *t*-test and non-parametric test were used to determine the preoperative and postoperative subjective scores. A non-parametric test was also used to determine the influencing factors of the VAS pain score. The differences between the two groups were analyzed by the *t*-test and non-parametric test. PASS 11.0 (NCSS, UT, USA) was used to calculate the sample size. The difference was considered significant at *p* < 0.05.

## Results

Of the 166 patients who met the inclusion criteria, 148 patients (89.2%) were available for the final follow-up, including 43 patients with OCL (group A) and 105 patients without OCL (group B). *Post hoc* power analysis showed that the sample size of 90 could achieve 95% power to detect the observed differences in the recovery of sports; thus, the sample of our research cases was enough. Preoperative demographic data and characteristics of two groups are compared in **Table 1**. The mean follow-up time of group A and group B was 63.6 ± 27.5 and 61.4 ± 21.0 months, respectively (n.s.). There was no significant difference in age, sex, injured side, types of ligament injury, and injured time (n.s.) between the two groups. The BMI of group A was significantly higher than that of group B (*p* < 0.05) (**Table 1**).

**Table 1:** Demographic data and characteristics of two groups.

	Group A (*n* = 43)	Group B (*n* = 105)	*p*-Value
Sex			0.697
Male	31	71	
Female	12	34	
Age (years)	29.1 ± 9.3	26.3 ± 10.7	0.052
BMI (kg/m^2^)	24.8 ± 3.1	23.4 ± 3.5	0.009*
Injury time (days)	8.8 ± 5.0	7.0 ± 3.5	0.065
Follow-up time (months)	63.6 ± 27.5	61.4 ± 21.0	0.828
Side			0.718
Left	24	62	
Right	19	43	
Types of ligaments injury			0.331
ATFL + CFL repair	33	84	
ATFL repair	8	21	
CFL repair	2	0	

* *Statistically significant difference (p < 0.05).*

For patients in group A, the OCL was accounted for 25 (58%) in zone 4, 13 (30%) in zone 6, and 5 (12%) in zone 3. According to the Ferkle and Cheng staging system, there were 22 (51%) cases of stage B–C OCL and 21 (49%) cases of stage D–F OCL; osteophyte resection was performed in 10 (7%) and 5 (3%) patients in groups A and B, respectively, with no significant difference. A total of 7 (5%) and 15 (10%) patients underwent loose body removal in groups A and B, respectively. Two patients demonstrated OCLs in both the medial talus and the tibial plafond (**Table 2**).

**Table 2:** Lesions and treatment of two groups.

	Group A (*n* = 43)	Group B (*n* = 105)
OCL		
Zone 4	25	
Zone 6	13	
Zone 3	5	
Tibial plafond	2	
Ferkle and Cheng stage		
B–C	22	
D–F	21	
Osteophyte resection	10	5
Loose body remove	7	15
Types of ligament injury		
ATFL + CFL repair	33	84
ATFL repair	8	21
CFL repair	2	0

At the final follow-up, a total of 5 (3%) and 13 (9%) patients reported mild ROM restriction (<10°) in groups A and B, respectively; 7 (5%) and 10 (7%) patients experienced sprain recurrence in groups A and B, respectively. The postoperative VAS pain score, AOFAS score, and Tegner score were significantly improved from the preoperative level for group A and group B (*p* < 0.001). There was no significant difference in the preoperative VAS pain score, AOFAS score, or Tegner scores between the two groups (n.s.) (**Table 3**). The average postoperative satisfaction of patients in group B was slightly higher than that of patients in group A (83.8 ± 7.5% vs. 79.4 ± 11.1%), but the difference was not statistically significant. The postoperative exercise level of group A and group B recovered to more than 90% of the normal level (91.2% ± 11.2%, 90.9% ± 13.3%, respectively, n.s.). The average time to return to sports of group A and group B was 4.4 ± 1.0 and 4.4 ± 1.2 months after surgery, respectively (n.s.). Significant differences were found in the postoperational VAS pain score between the two groups. The patients with OCL showed higher postoperational VAS pain score (0.8 ± 1.7) than patients without OCL (0.3 ± 0.8) (*p* < 0.05).

**Table 3:** Comparison of the clinical evaluation of two groups.

	Group A (*n* = 43)	Group B (*n* = 105)	*p*-Value
Recovery of sports			
Percentage (%)	91.2 ± 11.2	90.9 ± 13.3	0.761
Time (months)	4.4 ± 1.0	4.4 ± 1.2	0.716
ROM restriction, *n*	5	13	0.934
Sprain recurrence, *n*	7	10	0.612
Satisfaction (%)	79.4 ± 11.1	83.8 ± 7.5	0.194
VAS pain			
Preoperation	6.0 ± 2.0	6.0 ± 1.3	0.388
Postoperation	0.8 ± 1.7	0.3 ± 0.8	0.027*
Pre–post changes	6.3 ± 2.9	5.8 ± 1.6	0.543
AOFAS			
Preoperation	28.6 ± 17.6	24.3 ± 8.2	0.352
Postoperation	97.5 ± 4.8	98.4 ± 3.7	0.278
Pre–post changes	68.8 ± 20.8	74.6 ± 9.0	0.135
Tegner			
Preoperation	1.0 ± 0.6	0.7 ± 0.6	0.356
Postoperation	4.3 ± 1.2	4.4 ± 2.2	0.116
Pre–post changes	3.3 ± 1.3	3.5 ± 1.0	0.145

* *Statistically significant difference (p < 0.05).*

According to the subgroup analysis of the VAS score of group A, the postoperative VAS score of patients with stage D–F OCL was significantly higher than that of patients with stage B–C OCL (1.3 ± 2.1 vs. 0.3 ± 0.9, *p* = 0.038). No significant difference was found in the other parameters (**Table 4**).

**Table 4:** Other influencing factors related to the VAS pain score.

	Preoperation	*p*-Value	Postoperation	*p*-Value	Pre–post changes	*p*-Value
Sex		0.363		0.617		0.259
Male	6.1 ± 1.9		0.8 ± 1.6		6.1 ± 2.6	
Female	6.5 ± 1		0.5 ± 1.2		6 ± 1.8	
Age (years)		0.288		0.154		0.567
<20	6.2 ± 2.5		1.8 ± 2.5		7.1 ± 3.9	
20–40	5.8 ± 1.9		0.6 ± 1.3		6.1 ± 2.7	
≥40	6.8 ± 0.8		0.5 ± 1.2		6.3 ± 0.8	
BMI (kg/m^2^)		0.228		0.185		0.428
<24	5.2 ± 2.3		1.1 ± 2.3		6.7 ± 3.9	
24–28	6.6 ± 0.8		0.4 ± 0.9		6.2 ± 1.2	
≥28	7.1 ± 2		1.3 ± 1.4		5.9 ± 2.9	
Side		0.812		0.292		0.812
Left	6 ± 1.9		0.7 ± 1.8		6.3 ± 2.9	
Right	6.1 ± 2.1		1 ± 1.6		6.5 ± 2.9	
Ferkle and Cheng stage		0.564		0.038*		0.364
B–C	6.3 ± 1.4		0.3 ± 0.9		5.7 ± 1.3	
D–F	5.9 ± 2.3		1.3 ± 2.1		6.8 ± 3.6	
Location		0.201		0.225		0.629
Zone 4	6.5 ± 1.5		0.6 ± 1.1		5.9 ± 1.4	
Zone 6	5.8 ± 2.1		0.9 ± 2.3		6.9 ± 3.4	
Zone 3	4.4 ± 2.9		2 ± 2.5		6.8 ± 6.1	

* *Statistically significant difference (p < 0.05).*

## Discussion

The most important finding of this study was that concurrent arthroscopic treatment of OCL and open anatomic ligament repair achieved good mid- to long-term results and could be a reliable procedure for patients after severe acute ankle sprains. Except that the pain was more pronounced than in the control group, there were no differences in other outcomes. Postoperative pain was positively correlated with the grade of OCL.

The present study indicated that no significant difference was found in most outcomes and returned to sports between the two groups. Simultaneous treatment of OCL does not affect the outcomes of open ligament repair for acute ankle sprains. However, the literature on CAI showed controversial results. Gregush et al. concluded that isolated lateral ligament repair had higher ankle and hindfoot scores compared to repair with concomitant treatment of an OCL (25), while other studies have shown that the association of OCL treatment did not affect the efficacy in the treatment of CAI up to 6 years after operation (26, 27). The result of the present research might be due to better regeneration potential and special characteristics of OCL in acute ankle sprain compared to those of CAI. In the present study, stage B–C OCL was found in 22 (51%) patients and stage D–F OCL was found in the other 21 (49%) patients, but no subchondral cyst was found. On the other hand, subchondral bone cystic degeneration and combined joint degeneration were commonly associated with CAI and negatively affected osteochondral regeneration (17, 18). In addition, during acute injury, the organism responded rapidly to activate repair capability within a few hours, and the inflammatory process was stimulated immediately to promote the regeneration of injured tissue (28).

The results also showed that the postoperative VAS pain score of OCL with arthroscopic treatment was higher than patients without OCL. The reason for the higher VAS pain score incidence in group A might be mostly due to the debridement or microfracture during the arthroscopic OCL treatment, which led to inflammation or the stimulation of subchondral bone tissue. The soft tissue and bone were reported as the major source of pain during OCL (29–31). In addition, bone marrow edema of subchondral bone often occurs caused by OCL, and BMS in the early stage may increase the risk of postoperative pain (32, 33). However, it should be noted that although there were differences in VAS scores between the two groups, they were both at a low level. A difference of less than 1.4 points might not have much clinical significance (34). In addition, patients with acute ankle sprain combined with OCL recovered 91.2 ± 11.2% of preinjury sports level, and the time to return to sport was 4.4 ± 1.0 months, which achieved similar clinical effects compared with previous studies on open ankle lateral ligament repair without OCL (35, 36). The results of the present study indicated that simultaneous arthroscopic OCL treatment and open ligament repair could be a reliable operation with good mid- to long-term results.

The subgroup analysis showed that stage D–F OCL (involving subchondral bone) caused more long-term pain than stage B–C OCL (not involving subchondral bone), which was similar to previous studies on CAI (37, 38). The results further indicated that the subchondral bone irritation was highly correlated with pain. Other reasons might be due to the lack of formation of hyaline cartilage. Fibrocartilage could be obtained after BMS treatment instead of hyaline cartilage, but the former does not have sufficient mechanical properties during exercise, thereby resulting in postoperative pain (13–15). However, radiography and MRI were not performed at the follow-up and should be further accessed in future studies.

The results of this study indicated that the medial (zone 4) and lateral (zones 3 and 6) OCL accounted for 58% and 30%, respectively, which was consistent with previous research (19). Clinical outcomes in this study were not affected by the location of OCL. In CAI, medial lesions were demonstrated to be more common as well as larger in depth and surface area, so the recovery of lateral OCL was reported to be better than medial OCL (39–41). However, according to the present study, there was no significant difference in clinical results between the acute ankle sprains with medial and lateral OCL. The reason for similar clinical outcomes between different locations might be mostly that the severity of the lesion did not vary with the location of OCL in the present research. In addition, the articular surface injury of the corresponding tibial plafond was also reported to negatively affect the postoperative recovery (42), but only two cases were complicated with tibial OCL in the current study and had little effect on the outcome. A study with more cases was needed in the future.

This study also found that the BMI of patients with OCL was significantly higher than that of patients without OCL. Higher BMI was associated with higher absolute tibiofemoral compression (43, 44). In addition, a motion analysis study showed that in obese patients, the disproportionate stress is borne by force scattered in a small part of the whole articular surface (45). Biomechanical gait studies of the knee also showed that obese patients spent more time standing and knee weight-bearing (46). In addition, BMI was also found to be higher in patients with OCL than in patients without OCL in chronic lateral ankle instability (47). Therefore, patients with higher BMI were considered to have a greater possibility of OCL, but more in-depth research was needed in the future.

It should be noted that the patients of the two groups underwent different postoperative rehabilitation procedures, which might affect the outcome. In fact, a well-recognized rehabilitation program was used after ankle arthroscopy with or without OCL, rather than deliberately using different rehabilitation programs for the two groups of patients. Patients with OCL had CPM exercise 2 weeks after BMS to promote fibrocartilage regeneration by mechanical stimulus. Although the patients in group B did not have such training, they also underwent passive ankle ROM exercises daily from week 2 to avoid joint adhesion and resume the normal ROM. During the 2–3 weeks after the operation, except during the CPM activity, the ankle joint was still in a fixed state, so the immobilization time of the two groups was basically the same and the two groups were comparable. In terms of the weight-bearing time, the postoperative weight-bearing of patients undergoing OCL in group A was delayed by about 4 weeks compared with patients without OCL. Although some studies proposed immediate weight-bearing after BMS (6, 48), the delayed weight-bearing for postoperative cartilage procedure was more widely accepted to reduce postoperative pain and promote cartilage regeneration (49). In addition, our research focused on the mid- to long-term clinical effect and have a relatively long time from postoperative rehabilitation, so we proposed that the difference in conclusion mainly comes from OCL treatment itself but not the rehabilitation program.

To our knowledge, this is the first study to compare the long-term outcome of the acute ligament repair with or without OCL treatment. The follow-up evaluation included comprehensive parameters including recovery of sports, subjective score, ROM, and recurrence of sprain. The results offered insight into the effect of the OCL treatment on the acute ankle sprain outcomes and indicated a possible increased risk of VAS pain score in patients with stage D–F OCL, which should be informed to the patients before operations.

There are still some limitations to this research. First, it was retrospective rather than prospective research, and selection bias might affect the results. In addition, there was no stress x-ray or MRI examination to evaluate the objective ankle stability, OCL fate, and joint degeneration, which could be further accessed in future studies.

## Conclusion

Concurrent arthroscopic treatment of OCL and open anatomic ligament repair achieved good mid- to long-term results and could be a reliable procedure for patients after a severe acute ankle sprain. Except that the pain was more pronounced than in the control group, there were no differences in other outcomes. Postoperative pain was positively correlated with the grade of OCL.

## Data Availability

The raw data supporting the conclusions of this article will be made available by the authors without undue reservation.
